# Serum Progesterone Levels with The Use of Four Different Types of Vaginal Progesterone in Frozen-Thawed Embryo Transfer Cycles and Related Pregnancy Outcomes

**DOI:** 10.22074/IJFS.2021.6235

**Published:** 2021-01-27

**Authors:** Reiko Shiba, Masayuki Kinutani, Shinichiro Okano, Maki Ikeda, Emi Fukunaga, Yoshihisa Harada, Reo Kawano, Yuko Kikkawa

**Affiliations:** 1 Kinutani Women’s Clinic, 4F 8-23 Hondori, Naka-ku, Hiroshima-city, Hiroshima-Prefecture, Japan; 2 Center for Integrated Medical Research, Hiroshima University Hospital, Kasumi, Minami-ku, Hiroshima, Japan; 3 Katsuki Ladies Clinic, Hiroshima Central Street, Mikawa-cho, Naka-ku, Hiroshima-city, Hiroshima, Japan

**Keywords:** Embryo Transfer, Luteal Phase, Pregnancy Outcome, Serum Progesterone, Vitrification

## Abstract

**Background:**

Luteal phase support (LPS) is essential for hormone replacement therapy (HRT) for frozen-thawed embryo transfer (FET). However, the optimal dose and serum progesterone (P4) levels required for pregnancy are controversial. We attempted to determine the association between pregnancy outcomes and serum P4 levels adminis- tered via vaginal suppository for HRT-FET cycles on embryo transfer day.

**Materials and Methods:**

This was a secondary analysis of the dataset from the EXCULL trial, which prospectively investigated pregnancy outcomes of four different P4 vaginal suppositories (Lutinus, Utrogestan, Luteum, and Crinone) for HRT-FET. It was conducted at a private fertility clinic between December 2016 to December 2017. During this trial, 235 cycles were divided into four groups based on serum P4 values (quartile [Q] 1 group: <7.8 ng/mL; Q2 group: 7.8-10.8 ng/mL; Q3 group: 10.8-13.7 ng/mL; Q4 group: >13.7 ng/mL). We investigated clinical pregnancy rate (CPR), positive fetal heart rate (FHR), live birth rate (LBR), and miscarriage rate (MR) for each group. A logistic regression analysis was performed using age, body mass index (BMI), and transferred embryos as covariates.

**Results:**

Serum P4 values (ng/mL) of each drug were as follows: Lutinus, 13.3 ± 4.9; Utrogestan, 9.3 ± 3.3; Luteum, 13.6 ± 4.2; and Crinone, 8.7 ± 3.2 (mean ± standard deviation, P<0.001).The percentages of Utrogestan and Crinone were higher in the Q1 group, while the percentages of Lutinus and Luteum were higher in the Q4 group. Nonetheless, there were no statistical differences between the Q1 and Q4 groups in CPR, FHR, LBR, and MR.

**Conclusion:**

When vaginal P4 was used for FET, although serum P4 levels on transfer day differed based on the drug that was administered, no relationship was observed between serum progesteronelevels and pregnancy outcomes (Reg- istration number: UMIN000032997).

## Introduction

Currently, there is a growing preference for the use of frozen-thawed embryo transfer (FET), rather than fresh embryo transfer (fresh ET) (1). This is because FET may have a higher pregnancy rate as the hormonal status of the endometrium provides an environment that is suitable for implantation (2, 3), and also because ovarian hyperstimulation syndrome can be avoided (4). FET involves two methods: the use of the natural ovulation cycle (natural embryo transfer [N-ET]), as well as the use of the hormone replacement cycle (hormone replacement therapy [HRT] FET [HRT-FET]). HRT-FET provides a better management schedule; although, the replacement of external estrogen and progesterone (P4) is required until at least week 10 of pregnancy, at which point hormone production by the placenta should be sufficient (5-7). The required P4 replacement can be administered intramuscularly, orally, or transvaginally. However, vaginal suppositories are increasingly favored worldwide due to their ease of use and effective drug delivery to the endometrium of the uterus (8).

Currently, four different P4 vaginal suppositories are available for use: Lutinus (Ferring Pharmaceuticals, Saint-Prex, Switzerland), Utrogestan (FUJIFILM Phar-maceuticals, Japan), Luteum (ASKA Pharmaceutical, Japan), and Crinone (Merck Serono, Germany). The dosage and administration route of each drug have been determined through clinical trials of fresh ET (9-12). All four of these suppositories have been reported to be effective for fresh ET (13). Because FET has been recently developed, there are insufficient data regarding the optimal P4 dosage for each of these four vaginal suppositories to successfully induce clinical pregnancy. Moreover, the clinical significance of measuring serum P4 levels for FET remains to be established (5). Therefore, some infertility facilities increase the dose of the drug when the serum P4 level is low.

Recently, several studies have reported an association between serum P4 levels and pregnancy outcomes with HRT-FET and have shown a correlation between poor pregnancy outcomes and low serum P4 levels (14-17). From December 2016 to December 2017, we conducted a prospective, randomized, controlled study (the exploratory test) to investigate the clinical pregnancy rates (CPR) after using four types of vaginal P4 medications, Lutinus vaginal tablet, Utrogestan vaginal capsule, Luteum vaginal suppository, and Crinone vaginal gel (the EXCULL study). Outcomes of this trial indicated that the CPR, ongoing pregnancy rate (OPR), and miscarriage rate (MR) among the four suppositories were not significantly different (18). Therefore, we performed a secondary analysis of this data and examined the association between serum P4 levels on embryo transfer day and CPR, positive fetal heart rates (FHR), live birth rates (LBR), and MR for HRT-FET. 

## Materials and Methods

### Study design

This was a secondary analysis of data from our previously published EXCULL trial (18). The EXCULL study was a randomized, controlled trial that prospectively investigated the pregnancy outcomes of four different P4 vaginal suppositories (Lutinus, Utrogestan, Luteum, and Crinone) for HRT-FET. Since this study was an exploratory study, no particular sample size was established. Patients with contraindications listed on the medication package were excluded. This was the only exclusion criterion. We surveyed the number of patients that can be assigned to our clinic in one year.

Patients underwent egg retrieval at our hospital. Embryos were cryopreserved using vitrification, followed by transfer using a single cleavage-stage embryo or a blastocyst derived from the patient’s own egg. The HRT protocol was started with administration of transdermal estrogen (E2) tape (Estrana tape; Hisamitsu Pharmaceutical Co., Inc., Tosu, Saga Prefecture, Japan) or E2 gel (L’estrogel 0.06%; FUJIFILM Pharmaceuticals, MA), and its dosage was gradually increased. Luteal phase support (LPS) was then achieved using one of the four vaginal P4 suppositories. The four types of P4 suppositories were administered as follows: Lutinus, 100 mg three times daily; Utrogestan, 200 mg three times daily; Luteum, 400 mg two times daily; and Crinone, 90 mg once daily.

During this analysis, all 235 cycles included in the EXCULL study were analyzed. Endpoints were observation of a gestational sac at 5 weeks of gestation using transvaginal ultrasound (which was defined as CPR), positive FHR at 7 weeks of gestation, LBR, and MR.

### Blood sampling

Day (D) 0 represents the date on which the P4 vaginal suppository was initiated for LPS. Blood samples to quantify serum P4 levels were collected on D2, D3, or D5, which corresponded to the embryo transfer dates, from patients undergoing cleavage-stage embryo or blastocyst transfer, respectively. All blood samples were obtained an average of 4 hours after administration of the last vaginal suppository. The P4 concentration was measured at the hospital using an electro-chemiluminescence immunoassay (Cobas e 411 analyzer; Roche Diagnostics GmbH, Germany) according the manufacturer’s instructions. The detection limit of P4 was 0.0337 ng/mL.

### Statistical analysis

Pairwise comparisons among the four groups were performed using the analysis of variance (ANOVA) or Kruskal-Wallis test for continuous variables and the Chi-squared test for categorical variables. Associations between serum P4 levels and CPR, FHR, LBR, and MR were first analyzed by simple logistic regression analysis and then by multiple logistic regression analysis. The variables included were serum P4 levels, age, body mass index (BMI), pregnancy history, endometrial thickness, previous transfers, participation in the study, type of transferred embryos, and quality of blastocyst. Variables showing statistical significance were included as adjusting potential confounders in the multiple logistic regression analysis. The Hosmer-Lemeshow test was used to assess the goodness-of-fit of the logistic regression model. All statistical analyses were performed using statistical software (SAS 9.4; SAS Institute Inc., Cary, NC, USA). The level of significance was set at P<0.05.

### Ethical considerations

The EXCULL study was approved by the Ethics Committee of Kinutani Women’s Clinic (ethical review number: 2016-1110-1) and was registered with the Clinical Trials Registry (registration number: UMIN000032997). All women who agreed to participate in the study provided informed written consents.

## Results

The 235 cycles involved in the EXCULL study were divided into four groups with the lowest serum P4 levels on the embryo transfer day as follows: quartile (Q) 1 group, <7.8 ng/mL; Q2 group, 7.8-10.8 ng/mL; Q3, 10.8-13.7 ng/mL; and Q4 >13.7 ng/mL. Table 1 shows the baseline characteristics of all four groups (Table 1). The four groups did not differ with regard to age, BMI, pregnancy history, endometrial thickness, number of previous transfers, number of study participants, transferred embryos, or blastocyst quality. However, the proportion of vaginal agent used was significantly different. For Q1 group, the following rates were found: Lutinus, 10.2%; Utrogestan, 42.4%; Luteum, 5.1%; and Crinone, 42.4%. For Q2 group, the rates were as follows: Lutinus, 23.7%; Utrogestan, 27.1%; Luteum, 15.3%; and Crinone, 33.9%. For Q3 group, the rates were as follows: Lutinus, 25.4%; Utrogestan, 25.4%; Luteum, 35.6%; and Crinone, 13.6%. For Q4 group, the following rates were found: Lutinus, 48.3%; Utrogestan, 6.9%; Luteum, 39.7%; and Crinone, 5.2% (P<0.001).

**Fig.1:**
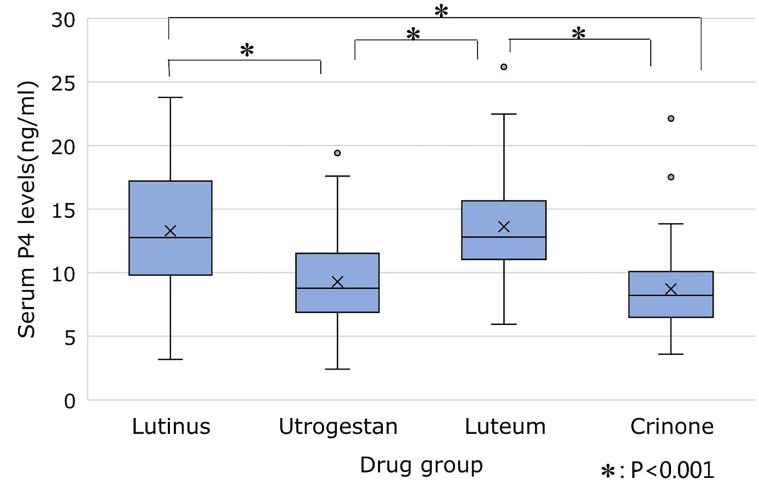
Box whisker plot depicting the serum progesterone levels (ng/mL) for each drug on embryo transfer day. * ; P<0.001.

The proportions of Utrogestan and Crinone were high in Q1 group, and those of Lutinus and Luteum were high in Q4 group. Because the ratios of drugs differed in Q1 and Q4, serum P4 values for each drug were confirmed (Table 2, Fig .1). P4 levels (mean ± standard deviation [SD]) were lower for Utrogestan (9.3 ± 3.3 ng/mL) and Crinone (8.7 ± 3.2 ng/mL) than for Lutinus (13.3 ± 4.9 ng/mL) and Luteum (13.6 ± 4.2 ng/mL) (P<0.001). A logistic regression analysis was used to detect an association among the four P4 groups and CPR, FHR, LBR and MR. In performing the logistic regression analysis, Hosmer-Lemeshow was used to evaluate the fitness of the regression model for each pregnancy outcome. We selected age, BMI, and transferred embryo as covariates for the multiple logistic regression analysis, because these factors had affected pregnancy outcomes (CPR, FHR, LBR) consistently and significantly in simple logistic regression analysis. Regarding MR, none of the variables showed statistical significance due to the small number of data. However, it was assumed that the same confounder as CPR, FHR, and LBR existed, so, it was adjusted with the same covariates. After adjusting for confounding factors (age, BMI, and type of transferred embryo), no association among the four groupsand CPR, FHR, LBR, and MR was identified (Table 3).

**Table 1:** Demographic characteristics and laboratory parameters of the patients


Serum P4 group		Q1 (<7.8 ng/mL)	Q2 (7.8-10.8 ng/mL)	Q3 (10.8-13.7 ng/mL)	Q4 (>13.7 ng/mL)	P value
		n=59	n=59	n=59	n=58	

Age (Y)		35.3 ± 4.5	36.1 ± 4.0	37.3 ± 4.0	36.6 ± 4.6	0.09^1^^†^
BMI (kg/m^2^)		20.5 ± 2.9	20.9 ± 2.4	20.8 ± 3.1	20.1 ± 1.8	0.361^†^
Drug						<0.001^§^
	Lutinus	6 (10.2)	14 (23.7)	15 (25.4)	28 (48.3)	
	Utrogestan	25 (42.4)	16 (27.1)	15 (25.4)	4 (6.9)	
	Luteum	3 (5.1)	9 (15.3)	21 (35.6)	23 (39.7)	
	Crinone	25 (42.4)	20 (33.9)	8 (13.6)	3 (5.2)	
Pregnancy history						0.768^§^
	Primary	26 (44.1)	26 (44.1)	31 (52.5)	27 (46.6)	
	Secondary	33 (55.9)	33 (55.9)	28 (47.5)	31 (53.4)	
Endometrial thickness (mm)		11.1 ± 1.7	11.1 ± 2.2	11.2 ± 1.9	11.1 ± 1.7	0.992^†^
Previous transfers						0.468^§^
	0	25 (42.4)	19 (32.2)	16 (27.1)	15 (25.9)	
	1	12 (20.3)	16 (27.1)	17 (28.8)	20 (34.5)	
	≥ 2	22 (37.3)	24 (40.7)	26 (44.1)	23 (39.7)	
Participation of study						0.563^§^
	1	45 (76.3)	42 (71.2)	36 (61.0)	38 (65.5)	
	2	9 (15.3)	14 (23.7)	18 (30.5)	15 (25.9)	
	≥ 3	5 (8.5)	3 (5.1)	5 (8.5)	5 (8.6)	
Transferred embryo						0.132^§^
	Cleavage-stage	28 (47.5)	32 (54.2)	31 (52.5)	20 (34.5)	
	Blastocyst	31 (52.5)	27 (45.8)	28 (47.5)	38 (65.5)	
Quality of blastocyst						0.781^§^
	High	21 (67.7)	21 (77.8)	21 (75.0)	26 (68.4)	
	Poor	10 (32.3)	6 (22.2)	7 (25.0)	12 (31.6)	
Clinical pregnancy rate		17 (28.8)	22 (37.3)	19 (32.2)	25 (43.1)	0.394^§^
Fetal heart beat rate		14 (23.7)	19 (32.2)	16 (27.1)	18 (31.0)	0.731^§^
Live birth rate		12 (20.3)	19 (32.2)	15 (25.4)	16 (27.6)	0.530^§^
Miscarriage rate		5 (29.4)	3 (13.6)	4 (21.1)	9 (36.0)	0.340^§^


BMI; Body mass index, P4; Progesterone, ^†^ ; ANOVA, and ^§^ ; Pearson's chi-square test, Q1-Q4 groups were divided according to the serum P4 level. Data are presented as mean ± SD or as number and count (%).

**Table 2:** Serum progesterone (P4) levels for each drug on the embryo transfer day


Drug	Lutinus n=63	Utrogestan n=60	Luteum n=56	Crinone n=56	P value

P4 levels (ng/mL)	13.3 ± 4.9	9.3 ± 3.3	13.6 ± 4.2	8.7 ± 3.2	<0.001


Data are presented as mean ± SD

**Table 3:** Logistic regression analysis of reproductive outcomes for the four progesterone groups


Type of analysis		Unadjusted analysis		Adjusted analysis	
		OR (95% CI)	P value	OR (95% CI)	P value

Clinical pregnancy					
	Q1	1		1	
	Q2	1.47 (0.68-3.18)	0.329	1.85 (0.81-4.22)	0.144
	Q3	1.17 (0.54-2.57)	0.689	1.63 (0.70-3.78)	0.259
	Q4	1.87 (0.87-4.03)	0.109	2.02 (0.89-4.57)	3
Fetal heart beat					
	Q1	1		1	
	Q2	1.53 (0.68-3.44)	0.307	1.92 (0.82-4.52)	0.135
	Q3	1.20 (0.52-2.74)	0.673	1.61 (0.67-3.90)	0.288
	Q4	1.45 (0.64-3.28)	0.377	1.56 (0.66-3.638)	0.311
Live birth					
	Q1	1		1	
	Q2	1.86 (0.81-4.29)	0.146	2.36 (0.98-5.71)	0.056
	Q3	1.34 (0.56-3.17)	0.512	1.79 (0.72-4.46)	0.214
	Q4	1.49 (0.63-3.51)	0.36	1.59 (0.65-3.89)	0.308
Miscarriage					
	Q1	1		1	
	Q2	0.38 (0.08-1.88)	0.236	0.33 (0.06-1.75)	0.193
	Q3	0.64 (0.14-2.92)	0.565	0.59 (0.11-3.10)	0.537
	Q4	1.35 (0.36-5.08)	0.657	1.17 (0.30-4.60)	0.822


Data shows OR of groups Q2, Q3, and Q4 when Q1 is set to 1. Adjusted variables: age, body mass index, and transferred embryo.

## Discussion

We determined whether P4 levels measured on embryo transfer day were related to pregnancy outcomes involving the use of four vaginal P4 suppositories for LPS. We did not identify an association among serum P4 levels and CPR, FHR, LBR and MR.

As previously noted, recent studies have reported that low P4 levels are associated with poor pregnancy outcomes (14-17). However, this study showed the opposite results. We identified three studies that specifically assessed the use of Utrogestan. The first of these was a prospective study of patients who underwent donor egg transfer and Utrogestan (400 mg) administered twice daily. In that study, CPR and OPR decreased when serum P4 levels on the embryo transfer day were <9.2 ng/mL (14). Because we did not use the same protocol for Utrogestan administration, a direct comparison of these findings with those of our study was not feasible. The area under the curve (AUC) of the receiver-operating characteristic (ROC) curve in that previous study was 0.59, indicating that P4 levels had a minimal ability to predict OPR. The second study of Utrogestan was a retrospective evaluation of patients who underwent euploid embryo transfer and preimplantation genetic testing for aneuploidies and were administered Utrogestan at a dose of 200 mg, three times daily (15). When P4 levels were <8.06 ng/mL on the day of embryo transfer, although there was no difference in CPR, the LBR decreased and the MR increased. The finding that the P4 level did not affect CPR is consistent with our findings in the current study. However, they did not proceed with embryo transfer if the P4 level was <5 ng/mL, which was different from our study. Therefore, comparisons between the results of that study and those of ours are not appropriate. The third study was a retrospective evaluation of patients receiving 200 mg of Utrogestan three times daily (16). In that study, serum P4 levels were <10 ng/mL before the transfer date led to lower CPR, OPR, and LBR. Moreover, these studies showed that when the P4 level was <10 ng/mL, increasing the dose to 400 mg three times daily failed to modify the pregnancy rate. However, the AUC in that study was only 0.62, indicating that the P4 level has low predictive ability for pregnancy outcomes. One retrospective study assessed the outcomes of blastocyst transfer with the administration of 90 mg of Crinone three times daily (17). CPR and OPR decreased when serum P4 levels on the pregnancy determination date were <35 nmol/L (approximately 11.1 ng/mL) while the 50th percentile of P4 levels was 34 nmol/L (10.8 ng/mL). Of note, despite the fact that the dose of Crinone was three folds higher than that used in our study, there was minimal difference from our mean P4 level of 8.7 ng/mL.

Our findings revealed differences in serum P4 levels across the four different types of P4 vaginal suppositories, however, all four drugs had a median P4 level of approximately 10 ng/mL and an overlap in their respective interquartile ranges. All four medications contain natural progesterone, but differ regarding the daily recommended dose: Crinone, 90 mg/day; Lutinus, 300 mg/day, Utrogestan, 600 mg/day; and Luteum, 800 mg/ day. Despite these large differences in daily dosages, the difference in measured serum P4 levels was with in 5 ng/ mL. Possible reasons include differences in the solubility and absorption rates of the drugs. Specifically, Lutinus and Crinone may have better absorption despite their lower P4 content. The measured P4 levels for these four types of suppositories in our study did not vary greatly from previously reported levels (14-17). With the dosage and usage currently suggested by drug manufacturers, all four drugs are anticipated to produce a serum P4 level of approximately 10 ng/mL.

A systematic review of previous studies regarding the use of vaginal P4 for *in vitro* fertilization cycles indicated the efficacy and safety of using Crinone, Lutinus, Utrogestan, and Luteum for fresh ET (13). However, the effective dosage and usage of vaginal suppositories for HRT-FET remain unclear due to insufficient data (5). Shapiro et al. (5) stated that monitoring serum P4 levels is ineffective due to the uterine first-pass effect, wherein the transvaginal administration of these drugs yields sufficiently higher endometrial concentrations than intramuscular injection despite the low serum P4 levels (19-21). 

In this study, the lowest serum P4 level that could result in a live birth was 4.06 ng/mL, and there were only three cycles with P4 levels <4 ng/mL. We also showed that the AUC of the ROC curve predicting CPR was 0.58, suggesting that serum P4 levels were poorly predictive of CPR.

We have shown in previous studies that there is no difference in pregnancy outcome when the four aforementioned drugs are used (18). In this study, four groups were created based on serum P4 levels, and pregnancy outcomes were examined. The P4 values of the four medications differed, but there was no difference in pregnancy outcomes among the groups. Therefore, there is no need to increase or decrease the medicine based on the serum P4 level, when each of the four drugs were used for HRT-FET as currently indicated by each pharmaceutical company. Although, the dosage and administration level were determined after a trial of fresh-ET. An advantage of our study is that it is a prospective trial with no patient selection bias. We also did not interrupt the study nor did we increase the amount of each drug with a low blood P4 level. High blood P4 levels may lead to drug leaks into the blood vessels instead of the endometrium, so the exclusion of patients with low P4 might be wrong.

This study had some limitations. The data used in our analysis were obtained from a single clinic in Japan, therefore, a patient bias effect cannot be denied. Furthermore, only Japanese patients were included. Compared to women in western cultures, Japanese women tend to have a lower BMI. Therefore, they require a lower dosage of P4 supplementation to attain target levels. In addition, because the serum P4 values of all the drugs are concentrated at approximately 10 ng/mL, it is not known whether there is an optimal serum P4 value that allows pregnancy. Furthermore, this is an exploratory study and we did not set the sample size, so increasing the sample size might make a significant difference in the pregnancy rates among the four groups. Furthermore, large-scale, multicenter, prospective cohort studies are needed to elaborate our findings.

## Conclusion

When vaginal P4 was used for FET, despite the fact that serum P4 levels on transfer day differed amongst the experimental groups based on the administered drug, no relationship was observed between serum progesterone levels and pregnancy outcomes. However, the relationship between serum P4 levels and pregnancy outcomes is still controversial and requires further studies.
